# From entrepreneurship education to entrepreneurial intention: Mindset, motivation, and prior exposure

**DOI:** 10.3389/fpsyg.2023.954118

**Published:** 2023-02-20

**Authors:** Junhua Sun, Jingyi Shi, Junfeng Zhang

**Affiliations:** ^1^School of Education, Nanjing University, Nanjing, China; ^2^Department of Mathematics and Statistics, Mississippi State University, Mississippi State, MS, United States

**Keywords:** entrepreneurship education, entrepreneurial intention, mindset, learning motivation, entrepreneurial exposure

## Abstract

We studied the relationships between entrepreneurship education and entrepreneurial intention among college students, with a focus on the mediating role of an entrepreneurial mindset as well as the moderating roles of learning motivation and prior entrepreneurial exposure. More than 90,000 students from 100 colleges or universities participated in the investigation, the data were subjected to structural equation modeling with Mplus. The results indicated that entrepreneurship education (curriculum attendance and extracurricular activity) significantly enhanced the entrepreneurial mindset of students, which, in turn, strengthened their entrepreneurial intention. In terms of learning, intrinsic motivation positively moderated the relationships between curriculum attendance and entrepreneurial intention/mindset, whereas extrinsic motivation moderated it negatively. Entrepreneurial exposure positively moderated the correlation between extracurricular activity and academic performance. Implications concerning the adjustment of entrepreneurship education to the entrepreneurial climate are discussed.

## Introduction

One of the highly debated topics in higher education that has attracted increasing attention in recent years relates to entrepreneurship education for college students (Santos et al., 2019; Cui et al., 2021; Wang et al., 2021). It has been of serious concern to administrators and course developers, researchers, and policymakers (Rauch and Hulsink, 2015), given the increasing importance of entrepreneurship in generating innovation and fueling economic growth (Sutter et al., 2019). Education in this field has the potential to boost the learning of college students and promote the acquisition of entrepreneurial knowledge, skills, and behavior (Cui et al., 2021), thereby enabling them to reach a high level of entrepreneurship (Jack and Anderson, 1999).

The aim of including entrepreneurship within higher education is to promote entrepreneurial intent and behavior among college students, and the entrepreneurial mindset has been deemed potential in terms of correlation (Solesvik et al., 2013; Pfeifer et al., 2016; Nabi et al., 2018; Cui et al., 2021). Rather than reflecting the *status quo*, it has been conceived of as a frame of mind for approaching problems, implementing innovations, finding solutions, sharing ideas, and making change happen, based on a spectrum associated with business ownership (Nadelson et al., 2018). It could be shaped through entrepreneurship education, not only in reflecting the thinking of entrepreneurs but also in enabling others to think and act like entrepreneurs. However, research on any link between entrepreneurship education and an entrepreneurial mindset, as well as on the role of the mindset in predicting entrepreneurial intention or behavior, is in the early stages (Krueger, 2015; Cui et al., 2021).

A meta-analysis carried out by Bae et al. (2014) revealed a significant but small correlation between entrepreneurship education and entrepreneurial intention, indicating the need to control for other variables that might affect the effectiveness of the education. Although learning motivation has been shown to affect the experiences of college students (National Survey of Student Engagement, NSSE, see Kuh, 2001), exactly how it benefits from entrepreneurship learning remains unclear (Hytti et al., 2010). In addition to curriculum attendance and extracurricular activity, prior entrepreneurial exposure has also been shown to boost entrepreneurial intention (Chlosta et al., 2012), such as by changing attitudes (Krueger et al., 2000; Zapkau et al., 2015). It would, therefore, be useful to assess learning motivation and entrepreneurial exposure as contextual factors of entrepreneurship education.

The following aspects are addressed in the current study. First, we investigate the impact of entrepreneurship education on the entrepreneurial mindset and the entrepreneurial intentions of college students. Despite the increasing number of empirical studies exploring entrepreneurship education, the entrepreneurial mindset, and entrepreneurial intention, a gap remains concerning how entrepreneurship education affects the two latter simultaneously. Thus far, results appear to be heterogenous (Cui et al., 2021), thereby leading to a lack of generality (Wang et al., 2021). Second, we examine the role of an entrepreneurial mindset in mediating the relationship between entrepreneurship education and entrepreneurial intention. Given the strengthening focus on the entrepreneurial mindset in validating entrepreneurship (Daspit et al., 2021), one should naturally acknowledge its power in explaining the mechanism by which entrepreneurship education affects entrepreneurial behaviors. Finally, we explore the role of learning motivation and entrepreneurial exposure in moderating the impact of entrepreneurship education on mindset and intention.

## Theoretical foundation and research hypotheses

### Entrepreneurship education and entrepreneurial intentions

Entrepreneurship education aims to develop students' entrepreneurial intentions (Li and Wu, 2019). As the optimal predictor of entrepreneurial behavior (Krueger et al., 2000), entrepreneurial intention has been highlighted in investigations of its relationship with entrepreneurship education (Zhang and Huang, 2021). Empirical studies have identified the mechanisms by means of which entrepreneurship education promotes entrepreneurial intentions (Nabi et al., 2018). First, *via* its courses and programs, it enables students to enhance their entrepreneurial knowledge, skills, attitudes, and even personal qualities (Wu et al., 2022). Second, incorporating field studies, internships, and extracurricular activities could give students entrepreneurial experience and constructive ideas. Finally, the process of learning could support the building of motivation and commercial networks, which could encourage student involvement (Egan et al., 2017).

Accordingly, our first hypothesis builds on findings showing how entrepreneurship education correlates with entrepreneurial behaviors (Ni and Ye, 2018) as follows:

**H1:** Entrepreneurship education relates positively to entrepreneurial intention.

### The mediating role of an entrepreneurial mindset

An entrepreneurial mindset reflects the capability to identify and exploit opportunities in the entrepreneurial field (Davis et al., 2016). It has been suggested that the surrounding environment paves the way for shaping mindsets (Zhang, 2022) through training or learning (Schmidt and Ford, 2003), thereby supporting the role of entrepreneurship education (Solesvik et al., 2013; Cui et al., 2021). As we understand it, this boosting functions in two ways: it creates an entrepreneurial climate at school, and it provides entrepreneur-related experience (Fayolle and Gailly, 2015).

Empirical evidence has shown that an entrepreneurial mindset is closely linked to individuals' entrepreneurial behavior (Lindberg et al., 2017), orienting behavioral patterns toward activities and outcomes related to entrepreneurship (Fayolle and Liñán, 2014). Thus, education has the potential to shape the mindset, which then predicts entrepreneurial intention. Accordingly, we posit that the entrepreneurial mindset could mediate between entrepreneurship education and entrepreneurial intention, as follows:

**H2:** Entrepreneurship education relates positively to the entrepreneurial mindset.
**H3:** An entrepreneurship mindset relates positively to entrepreneurial intention.
**H4:** An entrepreneurial mindset mediates the relationship between entrepreneurship education and entrepreneurial intention.

### The moderating role of learning motivation

Learning motivation is defined as the psychological will to drive and maintain behavioral patterns (Woolfolk, 2021) that may be generated by learning experiences and rewards. Motivation may be intrinsic or extrinsic (Biggs, 1987), the former referring to an internal intention in individuals to seek and overcome challenges, whereas the latter relates to external benefits, such as obtaining credits, avoiding punishment, and pleasing someone (Woolfolk, 2021). Among those with intrinsic motivation, the behavior itself gives internal and psychological pleasure without external reason or reward (Hytti et al., 2010), whereas extrinsic motivation relies on the environment to reap the rewards and thus is linked to negative emotions and maladaptive behaviors, at least to some extent (Vallerand et al., 1992).

Equally noteworthy is that extrinsic motivation has been reported to encourage individuals to try again, thereby enabling them to complete tasks that do not interest them (Woolfolk, 2021). In sum, according to cognitive theories, intrinsic motivation is more valuable than extrinsic, which is nevertheless necessary for maintaining motivation in general. Accordingly, we put forward the following hypotheses:

**H5a:** Intrinsic learning motivation positively moderates the relationship between entrepreneurship education and entrepreneurial mindset.
**H5b:** Extrinsic learning motivation negatively moderates the relationship between entrepreneurship education and entrepreneurial mindset.
**H6a:** Intrinsic learning motivation positively moderates the relationship between entrepreneurship education and entrepreneurial intention.
**H6b:** Extrinsic learning motivation negatively moderates the relationship between entrepreneurship education and entrepreneurial intention.

### The moderating role of prior entrepreneurial exposure

Nadelson et al. (2018) refer to “entrepreneurship on a spectrum, recognizing the contextual nature and psychological development associated with entrepreneurial thinking” (p. 114). This spectrum ranges from low to high in characteristics such as visionary thinking, creativity, self-regulation, risk-taking, resilience, and tolerance. According to Botha (2020, p. 2), “Aspiring entrepreneurs are more likely to start new businesses when they learn from existing entrepreneurs in the form of role models,” namely, in response to entrepreneurial exposure. Examples of entrepreneurial exposure include the experience of running a business or being an employer and contact with the family business or other entrepreneurial role models (Krueger, 2015). College students with entrepreneurial experience appear to have a higher level of entrepreneurial intention (Zapkau et al., 2015).

In sum, previous entrepreneurial exposure has the potential to moderate entrepreneurship education among college students. First, as an entrepreneurial learning experience (Sommarström et al., 2017), it could have a synergistic effect with other learning behaviors in enhancing academic performance; second, it could make those concerned more inclined to run a business in the future (Soria-Barreto et al., 2017), thereby boosting intrinsic motivation and adaptive entrepreneurial behaviors. Accordingly, we propose the following hypotheses:

**H5c:** Prior entrepreneurial exposure positively moderates the relationship between entrepreneurship education and an entrepreneurial mindset.
**H6c:** Prior entrepreneurial exposure positively moderates the relationship between entrepreneurship education and entrepreneurial intention.

**Figure 1:**
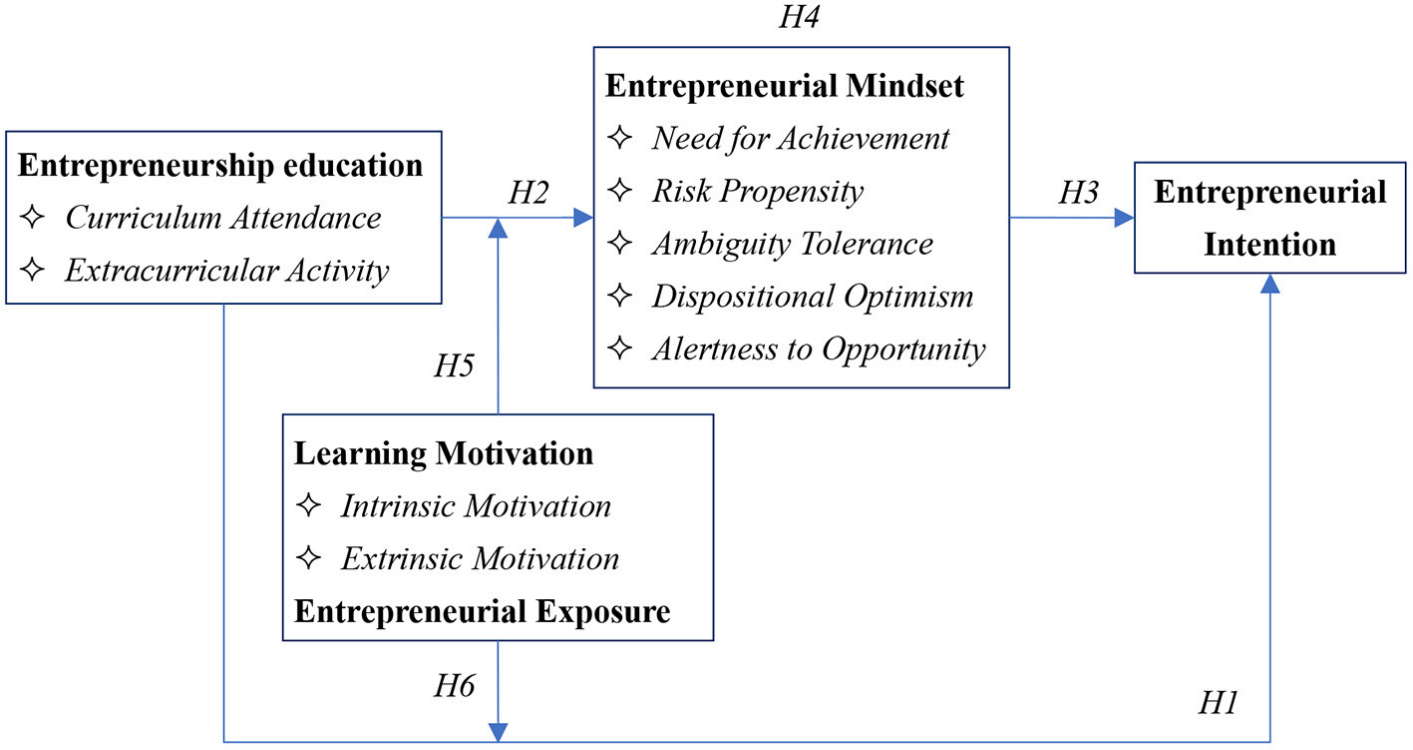
Theoretical and hypothesized model.

Figure 1 depicts our theory-based and hypothesis-based model.

## Materials and methods

### Participants and data collection

The participants were from more than 100 institutions of higher education in 28 provinces of China. The survey was carried out from December 2021 to January 2022 in the form of an online questionnaire; the participants' consent was obtained, and their information was anonymous. A pilot study was carried out in 2019 to adjust and revise the scale when more than 30,000 college students from 29 institutions of higher education completed the questionnaire. After eliminating invalid data, such as screening questions and unverified information from institutions, and given the limited filling-in time, we included a total of 92,284 questionnaires in the final data, representing a response rate of 75.11%. There were some missing values in a few variables; thus, the final sample for the hypothesis testing was 91510 (see Table 1, for the distribution of the validated sample).

**Table 1:** Distribution of the validated sample.

**Variable**	**Classification**	***N* of samples**	**Proportion (%)**
Gender	Males	39,564	42.87
	Females	52,720	57.13
Age	20 or less	74,848	81.18
	21 or more	17,353	18.81
Grade	Grade 2018	7,170	7.77
	Grade 2019	16,243	17.60
	Grade 2020	28,208	30.57
	Grade 2021	40,663	44.06
Major	Humanities & social science	18,661	20.22
	Economics & management	18,050	19.56
	Science & engineering	55,573	60.22
Educational level	Junior college	38,915	42.17
	Bachelor's degree	53,369	57.83

N = 92,284.

### Measures

#### Entrepreneurial intention

Based on a five-item questionnaire rated on a six-point Likert scale, this instrument was adopted from Chen et al. (1998). College students were asked to indicate how interested they were in and to what extent they were prepared to set up businesses in the future: the responses were graded on a scale ranging from 1 to 6, with six signifying the highest level.

#### Entrepreneurship education

Aimed at capturing participants' learning experiences of entrepreneurship, this instrument measured curriculum attendance and extracurricular activity. The measure of curriculum attendance, adopted from Sieger et al. (2014) and Cui et al. (2021), was based on one multiple-choice question concerning the number of entrepreneurial courses the college students had taken (0 for non-attendance and 1 for attendance). The measure of extracurricular activity (adopted from Arranz et al., 2017; Cui et al., 2021) was based on 10 items to assess the involvement of the participants in activities and events, such as the design competition, visits or internships, and talks by entrepreneurs, and the impact of these activities on them.

#### Entrepreneurial mindset

We focused on five factors. The need for achievement was measured on a five-item scale adopted by Zeffane (2013); tolerance of ambiguity and alertness to opportunity were measured on four items developed by Geller et al. (1993) and Tang et al. (2012), respectively, and risk propensity and dispositional optimism were measured on three items adopted by Hung et al. (2012) and Crane (2014), respectively.

#### Learning motivation and entrepreneurial exposure

Learning motivation was assessed on six items adopted by Hytti et al. (2010), three items for intrinsic and three for extrinsic motivation. Entrepreneurial exposure, adopted from Peterman and Kennedy (2003) and Botha (2020), concerned any experience the respondents had of starting or running a business, or if they had ever worked (including as an intern) in entrepreneurial business.

Table 2 lists the measurement instruments in some detail.

**Table 2:** The measurement items of the main variables.

**Variable**	**Measurement Item**	**Loading**
Entrepreneurial intention	(1) I am interested in setting up my own business	0.932
	(2) I have considered setting up my own business	0.964
	(3) I am preparing to set up my own business	0.970
	(4) I will try my best to set up my own business	0.934
	(5) I will set up my own business as soon as possible	0.924
Need for achievement	(1) I always do my best whether I am alone or with someone	0.940
	(2) I always try hard to improve on my past performance	0.944
	(3) I enjoy working toward clear and challenging goals	0.943
	(4) In general, I try to make every minute count	0.937
	(5) I often put pressure on myself to achieve as much as I can	0.911
Risk propensity	(1) I like to take chances, although I may fail	0.935
	(2) I like waiting until things have been tested before I try them out	0.913
	(3) I seek new experiences even if their outcomes may be risky	0.950
Ambiguity tolerance	(1) If I am uncertain about the responsibilities involved in a task, I get very anxious	0.894
	(2) It disturbs me when I am unable to follow another person's train of thought	0.839
	(3) Before doing any important task I must know how long it will take	0.930
	(4) A good task is one in which what is to be done and how it is to be done are always clear	0.927
Dispositional optimism	(1) In uncertain times, I would expect the best	0.913
	(2) I am always optimistic about my future	0.926
	(3) Overall, I expect more good things to happen to me than bad	0.934
Alertness to opportunity	(1) I have frequent interactions with others to acquire new information	0.921
	(2) I am keen on looking for information	0.910
	(3) I can recognize links between seemingly unrelated pieces of information	0.937
	(4) I can distinguish between profitable and non-profitable opportunities	0.936
Intrinsic motivation	(1) I am interested in studying entrepreneurship	0.984
	(2) I would study entrepreneurship even if I did not have to	0.982
	(3) Studying entrepreneurship is not useless because one day I may be an entrepreneur myself	0.983
Extrinsic motivation	(1) Because getting a good diploma is important, one should get good grades in entrepreneurship courses	0.962
	(2) Students of entrepreneurship must manage the courses and exams effectively	0.986
	(3) You have to study entrepreneurship to get a good job	0.987
Extracurricular activity	(1) Entrepreneurship clubs	0.894
	(2) Design competition	0.893
	(3) Listening to entrepreneurs' talks	0.869
	(4) Visit to an enterprise or an internship	0.875
	(5) Face-to-face communication with an entrepreneur	0.939
	(6) Conferences or workshops related to entrepreneurship	0.915
	(7) Business simulators or games	0.892
	(8) Entrepreneurial incubation project	0.954
	(9) Entrepreneurial activity involving resourcing or networking	0.959
	(10) Entrepreneurial spirit and values transmitted by the university or colleges	0.828

To avoid possible bias and to improve the reliability of the empirical results, we have included personal background information, including gender, age, grade, major, and educational level as control variables.

### Statistical analysis

We used SPSS 27.0 software to cleanse the data and Mplus 8.0 for the analysis. The first step was to conduct an exploratory factor analysis and a confirmatory factor analysis of two random half-samples to ensure reliability and validity. Second, we tested the hypotheses by means of structural equation modeling. Finally, we tested the mediating role and moderating effect as in Preacher et al. (2007).

## Results

### Reliability and validity

Table 3 gives the reliability, validity, and descriptive statistics for the variables in the research model. Based on Cronbach's alpha (α) and composite reliability (CR), the α values were above 0.8, ranging from 0.944 to 0.989, and the CR value was above 0.6, ranging from 0.943 to 0.989 (Bagozzi and Yi, 1988), indicating high reliability.

**Table 3:** Reliability, validity, correlation, and descriptive statistics.

**Variable**	**1**	**2**	**3**	**4**	**5**	**6**	**7**	**8**	**9**	**10**	**11**	**12**
1. Intention	*(0.945)*											
2. Mind_na	0.588	*(0.935)*										
3. Mind_rp	0.661	0.860	*(0.933)*									
4. Mind_at	0.540	0.837	0.777	*(0.898)*								
5. Mind_do	0.576	0.846	0.816	0.787	*(0.924)*							
6. Mind_ao	0.641	0.857	0.865	0.807	0.855	*(0.926)*						
7. Mindset	0.681	–	–	–	–	–	–					
8. Mov_i	0.411	0.349	0.369	0.333	0.338	0.372	0.393	*(0.983)*				
9. Mov_e	0.357	0.326	0.335	0.318	0.314	0.343	0.363	0.969	*(0.978)*			
10. Exposure	0.084	0.060	0.073	0.061	0.057	0.076	0.076	0.117	0.115	–		
11. Extra	0.533	0.368	0.431	0.364	0.366	0.431	0.448	0.460	0.431	0.136	*(0.903)*	
12. Curriculum	0.219	0.152	0.173	0.155	0.146	0.177	0.184	0.897	0.916	0.119	0.348	–
*Min*	1	1	1	1	1	1	1	0	0	0	0	0
*Max*	6	6	6	6	6	6	6	6	6	1	6	1
*Mean*	3.954	4.555	4.388	4.481	4.522	4.426	4.436	3.266	3.373	0.259	3.728	0.710
*SD*	1.460	1.088	1.164	1.099	1.123	1.125	1.050	2.326	2.353	0.438	2.251	0.454
α	0.976	0.972	0.952	0.944	0.946	0.960	–	0.989	0.986	–	0.978	–
*CR*	0.977	0.972	0.953	0.943	0.946	0.960	–	0.989	0.985	–	0.978	–
*AVE*	0.893	0.874	0.870	0.807	0.855	0.858	–	0.966	0.957	–	0.815	–
*N of items*	5	5	3	4	3	4	19	3	3	1	10	1

Intention, entrepreneurial intention; Mind_na, need for achievement; Mind_rp, risk propensity; Mind_at, ambiguity tolerance; Mind_do, dispositional optimism; Mind_ao, alertness to opportunity; Mindset, entrepreneurial mindset; Movi, intrinsic motivation; Move, extrinsic motivation; Exposure, prior entrepreneurial exposure; Extra, extracurricular activity; Curriculum, curriculum attendance. The italic values in parenthesis on the diagonal are the square roots of the AVE, and the values below the hypotenuse of the triangle are correlations among the variables.

N = 91,510. All correlations are significant at the 0.01 level.

The standardized coefficient loading of the items on the corresponding construct was significant (above 0.5 and ranging from 0.828 to 0.987, see Table 2), and the average variance extracted (AVE) values were higher than 0.5 (Bagozzi and Yi, 1988), indicating convergent validity for each variable (Fornell and Larcker, 1981). The square root of the AVE (the diagonal elements in Table 3) was larger than that of the off-diagonal elements at the level of significance (Hulland, 1999), indicating discriminant validity (Fornell and Larcker, 1981).

### Common method variance

We used Harman's single-factor method to test common method variance (Podsakoff et al., 2003). The percentage of variance explained by the first factor in the exploratory factor analysis was far below the threshold of 0.50. In the confirmatory factor analysis, the fitness of the single-factor model failed to meet the criteria (CFI = 0.448 < 0.90, TLI = 0.418 < 0.90, RMSEA = 0.221 > 0.08, SRMR = 0.195 > 0.08), the values of the variance inflation factor were <3, and the values of tolerability were more than 0.30. Therefore, common method variance did not affect the outcome of this study.

### Hypothesis testing

#### Correlation

Table 3 also shows the results of the correlation analysis, namely, the positive correlation (*p* < 0.01) between entrepreneurship education (curriculum attendance and extracurricular activity), entrepreneurial mindset, entrepreneurial intention, learning motivation, and entrepreneurial exposure. However, further analysis is necessary to test the validity of the research hypotheses.

#### Direct effect

Table 4 presents the results of the path analysis of the effect of entrepreneurship education on entrepreneurial intention and an entrepreneurial mindset. The path coefficients indicating the direct effects of (a) curriculum attendance and extracurricular activity on entrepreneurial intentions, (b) curriculum attendance and extracurricular activity on an entrepreneurial mindset, and (c) an entrepreneurial mindset on entrepreneurial intentions were all positive and significant (*p* < 0.001), thereby supporting H1, H2, and H3.

**Table 4:** Path analysis of the direct and indirect predictions.

**Variable**	**DV** = **Intention**		**DV** = **Mindset**	
	**Estimate**	**S.E**.	**Estimate**	**S.E**.
Intercept	2.081^***^	0.068	1.463^***^	0.075
EL	−0.138^***^	0.007	−0.117^***^	0.007
Gender	−0.214^***^	0.007	−0.055^***^	0.007
Age	−0.090^***^	0.003	0.145^***^	0.004
Grade	−0.027^***^	0.005	−0.207^***^	0.005
Major 2	0.146^***^	0.009	−0.200^***^	0.009
Major 3	0.120^***^	0.008	−0.094^***^	0.008
Curriculum	0.170^***^	0.009	0.128^***^	0.008
Extra	0.182^***^	0.002	0.190^***^	0.002
Mindset	0.718^***^	0.004		
*R^2^*	0.539	0.003	0.268	0.004
*F*	196.219^***^		75.464^***^	
*IND_C*	0.092^***^	0.006		
	[0.080, 0.104]			
*IND_E*	0.137^***^	0.001		
	[0.134, 0.139]			

Control variables are as follows: *EL* (educational level and junior college as reference), gender (female as reference), age, grade, major (humanities and social science as a reference, major 2 = economics and management, major 3 = science and engineering). IND_C/IND_E the mediating effect of entrepreneurial mindset from curriculum attendance/extracurricular activity to entrepreneurial intention.

N = 91,510. ^*^p < 0.05, ^**^p < 0.01, ^***^p < 0.001, two-tailed test. Numbers in [] are confidence intervals at the 95% level and bootstrapping n = 5,000.

#### Mediating effect

Path analysis and bootstrapping (*n* = 5,000) were used in the process of testing mediation (Preacher et al., 2007), as shown in Table 4. The indirect path to an entrepreneurial mindset from curriculum attendance and extracurricular activity was positive and significant (*p* < 0.001), and the bootstrapping confidence intervals at 95% were [0.080, 0.104] and [0.134, 0.139], which skip 0. Hence, the indirect path of an entrepreneurial mindset from entrepreneurship education to entrepreneurial intention was significant. The direct coefficients of entrepreneurship education were significant, too, confirming the role of an entrepreneurial mindset as a partial mediator. Therefore, H4 was supported.

#### Moderating effect

Table 5 presents the results of the tests for a moderating effect between learning motivation among college students and prior entrepreneurial exposure. The coefficients of the interaction connecting curriculum attendance and intrinsic learning motivation with entrepreneurial intention and an entrepreneurial mindset are positively significant, whereas those concerning extracurricular activity are negatively significant. Therefore, H5a and H6a are partially supported: in other words, intrinsic learning motivation among college students positively moderates the effect of curriculum attendance on entrepreneurial intention and mindset.

**Table 5:** Path analysis of the moderating effects.

**Variable**	**DV** = **Intention**				**DV** = **Mindset**			
	**Estimate**	**S.E**.	**Estimate**	**S.E**.	**Estimate**	**S.E**.	**Estimate**	**S.E**.
Intercept	2.226^***^	0.063	2.065^***^	0.067	1.565^***^	0.075	1.466^***^	0.075
EL	−0.138^***^	0.007	−0.132^***^	0.007	−0.107^***^	0.007	−0.113^***^	0.007
Gender	−0.196^***^	0.007	−0.210^***^	0.007	−0.038^***^	0.006	−0.051^***^	0.007
Age	−0.081^***^	0.003	−0.090^***^	0.003	0.140^***^	0.004	0.145^***^	0.004
Grade	−0.024^***^	0.005	−0.035^***^	0.005	−0.164^***^	0.005	−0.212^***^	0.005
Major 2	0.128^***^	0.009	0.145^***^	0.009	−0.196^***^	0.009	−0.201^***^	0.009
Major 3	0.102^***^	0.008	0.120^***^	0.008	−0.108^***^	0.008	−0.094^***^	0.008
Mindset	0.629^***^	0.005	0.717^***^	0.004				
Curriculum	−0.672^***^	0.025	0.197^***^	0.010	−2.033^***^	0.023	0.146^***^	0.009
Extra	0.194^***^	0.003	0.177^***^	0.002	0.154^***^	0.003	0.181^***^	0.002
Movi	0.232^***^	0.006			0.152^***^	0.005		
Move	−0.115^***^	0.006			0.106^***^	0.005		
Exposure			0.073^***^	0.022			−0.038	0.020
Curriculum* movi	0.232^***^	0.006			0.152^***^	0.005		
	[0.220, 0.243]				[0.141, 0.162]			
Curriculum* move	−0.115^***^	0.006			0.106^***^	0.005		
	[−0.127, −0.103]				[0.095, 0.116]			
Extra* movi	−0.034^***^	0.003			−0.007^***^	0.003		
	[−0.040, −0.028]				[−0.012, −0.002]			
Extra* move	0.024^***^	0.003			−0.003	0.003		
	[0.018, 0.030]				[−0.008, 0.003]			
Curriculum* exposure			−0.122^***^	0.021			−0.075^***^	0.020
			[−0.161, −0.081]				[−0.113, −0.035]	
Extra* exposure			0.020^***^	0.004			0.035^***^	0.004
			[0.013, 0.028]				[0.028, 0.042]	
*R^2^*	0.562	0.003	0.539	0.003	0.410	0.004	0.269	0.004
*F*	212.625^***^		196.566^***^		114.655^***^		75.957^***^	

N = 91,510. ^*^p < 0.05, ^**^p < 0.01, ^***^p < 0.001, two-tailed test. Numbers in [] are confidence intervals at the 95% level and bootstrapping n = 5,000.

The significance of the coefficients (*p* < 0.001) indicates that the interaction between curriculum attendance and extrinsic learning motivation is negatively related to entrepreneurial intention but positively related to an entrepreneurial mindset. Moreover, the interaction between extracurricular activity and extrinsic learning motivation is positively and significantly related to entrepreneurial intention but not significantly related to an entrepreneurial mindset. Therefore, H6b is partially supported, while H5b is not supported. In other words, extrinsic learning motivation among college students negatively moderates the relationship between curriculum attendance and entrepreneurial intention.

The coefficients of the interaction linking curriculum attendance and prior entrepreneurial exposure to entrepreneurial intention and an entrepreneurial mindset are negatively significant, whereas those of the interaction linking extracurricular activity and prior entrepreneurial exposure are positive and significant. Therefore, H5c and H6c are partially supported. In other words, previous entrepreneurial exposure positively moderates the predicted effect of extracurricular activity on entrepreneurial intention and an entrepreneurial mindset.

#### Robustness

Five dimensions of an entrepreneurial mindset were introduced as five mediators in the robustness tests, and the results are shown in Table 6. Without the change, the mediating effect was relatively robust, not only for an entrepreneurial mindset but also for the five dimensions.

**Table 6:** Robustness testing: The results of a mediating role.

**Variable**	**DV** = **Intention**				
CVs	Yes	Yes	Yes	Yes	Yes
Curriculum	0.193^***^	0.166^***^	0.198^***^	0.200^***^	0.183^***^
Extra	0.224^***^	0.217^***^	0.237^***^	0.225^***^	0.198^***^
Mind_na	0.580^***^				
Mind_rp		0.471^***^			
Mind_at			0.505^***^		
Mind_do				0.551^***^	
Mind_ao					0.613^***^
*R^2^*	0.484	0.476	0.447	0.477	0.506
*F*	173.656^***^	157.637^***^	158.621^***^	170.566^***^	178.956^***^16pt
**Variable**	**DV** = **Mind_na**	**DV** = **Mind_rp**	**DV** = **Mind_at**	**DV** = **Mind_do**	**DV** = **Mind_ao**
CVs	Yes	Yes	Yes	Yes	Yes
Curriculum	0.119^***^	0.204^***^	0.127^***^	0.113^***^	0.129^***^
Extra	0.164^***^	0.216^***^	0.163^***^	0.171^***^	0.197^***^
*R^2^*	0.177	0.532	0.153	0.153	0.244
*F*	65.642^***^	58.418^***^	63.191^***^	68.467^***^	73.815^***^
IND_C	0.069^***^	0.096^***^	0.064^***^	0.062^***^	0.079^***^
	[0.059, 0.079]	[0.086, 0.106]	[0.055, 0.073]	[0.052, 0.072]	[0.068, 0.090]
IND_E	0.095^***^	0.102^***^	0.082^***^	0.094^***^	0.121^***^
	[0.093, 0.098]	[0.099, 0.104]	[0.080, 0.084]	[0.092, 0.097]	[0.118, 0.123]

N = 91,510. ^*^p < 0.05, ^**^p < 0.01, ^***^p < 0.001, two-tailed test. Numbers in [] are confidence intervals at the 95% level and bootstrapping n = 5,000.

## Discussion

With regard to H1, the results showed correlations between entrepreneurship education and entrepreneurial intention, which is in line with existing empirical evidence both at home and abroad (Cui et al., 2021; Wang et al., 2021), namely, entrepreneurship education helps to equip students with the relevant knowledge and skills to take advantage of opportunities in the entrepreneurial field (Nadelson et al., 2018). Furthermore, related to H5 and H6 together, the above-mentioned experiences of learning and entrepreneurial exposure may enhance business success later on.

The results related to H2, H3, and H4 verified the role of a mediator in developing an entrepreneurial mindset, correlating with entrepreneurship education, and entrepreneurial intention. Growth orientation could develop through training and acquiring knowledge of entrepreneurship (Schmidt and Ford, 2003), boosting achievement needs, ambiguity tolerance, alertness, risk propensity, and dispositional optimism, for example. These psychological factors help college students to identify and exploit opportunities associated with business ownership (Davis et al., 2016). Again, according to the results related to H5 and H6, learning motivation moderates the correlations between entrepreneurship education and entrepreneurial intention: similar to mindset, it represents the psychological approach to behavioral patterns (Woolfolk, 2021). Thus, the educational environment should better embody growth-mindset principles and internal-motivation practices (Zhang, 2022).

In sum, creating an entrepreneurial learning climate facilitates the acquisition of entrepreneurship-related knowledge and skills on the one hand and encourages students to be self-efficient on the other, thereby supporting them in the business-startup process. We assume that the key issue of transiting the entrepreneurial environment relies on the shaping of implicit theories of entrepreneurial capability and the building of internal motivation.

### Implications

On the theoretical level, this study contributes to the literature on entrepreneurship education, especially from the perspective of educational psychology. First, the empirical study conducted among Chinese students verified the correlation between entrepreneurship education and entrepreneurial intention, thereby enriching the existing literature from Eastern cultures. Second, the findings acknowledge the supporting role of psychological aspects such as mindset and motivation in entrepreneurship education, verifying the importance of students' subjective initiatives in validating learning experiences. Third, earlier entrepreneurial exposure was introduced as a contextual factor, thereby reflecting the importance of the entrepreneurial climate in validating entrepreneurship education.

On the practical level, the findings offer insights that could help administrators in government and policymakers in institutions to develop entrepreneurship education further. First, they attest to the value of education in fostering innovativeness among students in higher education institutions. Thus, more attention in government and these institutions should be placed on supporting entrepreneurship education, such as in the funding and reinforcement of the curriculum. Second, mindset, motivation, and prior experience are advantageous to students in developing behavioral patterns related to entrepreneurship. Teachers should therefore be aware of what students enjoy in the learning process so that they can motivate them further and tailor relevant courses or activities to maximize the benefits.

### Limitations and future directions

The current study has several limitations that future research might draw on. First, given the major revision of the entrepreneurial mindset adopted in the current study from Dweck's original, there is a need to gather more empirical evidence to test the validity and generality of the five-item scale of the entrepreneurial mindset adopted from existing literature. Second, according to our findings, the moderating roles of learning motivation and entrepreneurial exposure varied with aspects of entrepreneurship education (curriculum attendance and extracurricular activity), but we failed to identify the underlying details. Future research should, therefore, focus on specific issues of education that may affect its impacts, such as curriculum planning, pedagogical strategies, and teaching materials. Third, given the tremendous sample size of our cross-sectional survey, although it thoroughly identified the correlations between entrepreneurship education and corresponding intentions *via* the mindset, it was not a scientifically causal analysis. A quasi-experiment comprising a randomized controlled trial or dynamic tracking would shed deeper light on the causal mechanism. Fourth, the empirical study was merely conducted among Chinese students; future research on patterns of entrepreneurial thinking that may be culture dependent should focus on the cross-cultural perspective, thereby tailoring education to improving the entrepreneurial climate.

## Conclusion

Using a cross-sectional survey of almost 100,000 college students, we examined the correlations between entrepreneurship education and the corresponding mindset and intention, including the mediating role of the entrepreneurial mindset and the moderating role of learning motivation and entrepreneurial exposure. The findings indicate that, first, entrepreneurship education, comprising curriculum attendance or extracurricular activities, directly predicts an entrepreneurial mindset and entrepreneurial intention. Second, entrepreneurship education predicts entrepreneurial intention indirectly by stimulating the mindsets of college students, verifying the mediating role of the entrepreneurial mindset. Third, intrinsic motivation positively moderates the relationships between curriculum attendance and entrepreneurial intention/mindset, whereas extrinsic motivation moderates them negatively. Fourth, previous entrepreneurial exposure among college students positively moderates the prediction of entrepreneurship education (particularly extracurricular activity) in terms of entrepreneurial intention. In addition to shedding light on the ordinary routine of entrepreneurship education, the results verify the importance of individuals' psychological initiatives and the learning climate in establishing adaptive behavioral patterns. The vital aspect of transiting the entrepreneurial environment may rely on the shaping of subjective entrepreneurial initiatives.

## Data availability statement

The original contributions presented in the study are included in the article/supplementary material, further inquiries can be directed to the corresponding author.

## Ethics statement

The studies involving human participants were reviewed and approved by the Ethical Review Board of the School of Education at Nanjing University, as well as the colleges or universities involved in the survey. All participants gave their informed consent. Written informed consent was not required.

## Author contributions

JSu designed the study, conducted the analysis, drafted the manuscript, and reviewed and revised the manuscript. JSh designed the study and reviewed the manuscript. JZ drafted, reviewed, and revised the manuscript. All authors approved the final submission and publication.
